# Direct and Vicarious Experiences of COVID-19-Related Racism Across Racial and Ethnic Groups in the United States

**DOI:** 10.1007/s40615-024-02159-x

**Published:** 2024-11-20

**Authors:** Yong Ju Cho, Juliana S. Sherchan, Jessica R. Fernandez, Sydney A. Barlow, Paula D. Strassle, Allana T. Forde

**Affiliations:** Division of Intramural Research, National Institute On Minority Health and Health Disparities, National Institutes of Health, Bethesda, MD USA

**Keywords:** COVID-19-related racism, Direct racism, Vicarious racism, Race and ethnicity, Racial and ethnic disparities

## Abstract

**Objectives:**

Due to stigmatization associated with the COVID-19 pandemic, certain groups were believed to be the cause of COVID-19 and thus experienced COVID-19-related racism through direct interpersonal and vicarious experiences. This study used quantitative and qualitative responses to examine whether the prevalence of experiencing these types of racism varied across racial and ethnic groups.

**Study Design:**

This cross-sectional study included 5,480 participants in the REACH-US (Race-Related Experiences Associated with COVID-19 and Health in the United States) study, which is a nationally representative survey administered to 5,500 U.S. adults from January 26, 2021-March 3, 2021.

**Methods:**

COVID-19-related racism was measured using single items about whether participants: 1.) experienced racism because they were thought to belong to a group more likely to get COVID-19 (direct); 2.) witnessed racism against others who were thought to belong to a group more likely to get COVID-19 (vicarious). Logistic regression examined differences in experiencing COVID-19-related racism across racial and ethnic groups, adjusting for sociodemographic characteristics. Themes emerged from open-ended descriptions of racism experiences coded in a thematic analysis and were reported across racial and ethnic groups.

**Results:**

Overall, 6.4% and 15.9% of adults experienced direct and vicarious COVID-19-related racism, respectively. All racial and ethnic groups (except Hispanic/Latino English Language Preference) were significantly more likely than White adults to experience direct (AORs: 2.06–4.92) and vicarious (AORs: 1.63–3.02) COVID-19-related racism. Racial and ethnic differences were observed across thematic domains of *type of mistreatment* and *settings where racism occurred*.

**Conclusions:**

Direct and vicarious COVID-19-related racism were more prevalent among marginalized racial and ethnic groups, comprised various types of mistreatment, and occurred across multiple settings, thus highlighting the need for integrated efforts to reduce and prevent racism.

**Supplementary Information:**

The online version contains supplementary material available at 10.1007/s40615-024-02159-x.

## Introduction

Throughout history, numerous infectious disease outbreaks have occurred that have not only contributed to significant burden, but to stigma that is often associated with discrimination against marginalized racial and ethnic groups [1]. Despite the knowledge gained from previous outbreaks (e.g., Human Immunodeficiency Virus, Ebola), the stigma associated with infectious disease outbreaks persists as evidenced by the most recent outbreak of SARS-Cov-2 (COVID-19).

During the COVID-19 pandemic, the stigma associated with COVID-19 stemmed from anti-Asian rhetoric tied to the origin of COVID-19. In addition, pre-existing prejudice and negative stereotypes contributed to an increase in discriminatory acts against marginalized racial and ethnic groups. The number of reported race-related hate crimes in the U.S. also surged, with a dramatic increase in anti-Asian (increased by 70%) and anti-Black (increased by 49%) hate crimes in 2020 [2]. Additionally, those belonging to marginalized racial and ethnic groups were significantly more likely than White adults to experience discrimination [3–6] or racism [7] throughout the COVID-19 pandemic.

Two types of discrimination or racism experiences—direct and vicarious—have been reported in the COVID-19 literature. Direct racism is defined as experiencing discriminatory actions through direct interpersonal interactions, whereas vicarious racism refers to witnessing discriminatory actions against other individuals [8]. Several previous studies focused on “any” discrimination or racism experiences specifically due to COVID-19 or COVID-19 stigma [1, 3, 6, 9–15]. Some studies focused solely on direct COVID-19-related discrimination or racism [3, 6, 11–13] and one study did not differentiate between direct and vicarious COVID-19-related discrimination or racism [1]. There were fewer studies that measured both direct and vicarious types of discrimination or racism [9, 10, 14, 15], and made comparisons across racial and ethnic groups [10, 14]. Among the studies on direct and vicarious COVID-19-related racism, most studies reported that individuals experienced more vicarious than direct COVID-19-related discrimination or racism [10, 14], and the Asian racial group had a higher prevalence of both direct and/or vicarious COVID-19-related discrimination. However, none of the studies examining both direct and vicarious COVID-19-related discrimination or racism, to our knowledge, used both quantitative and qualitative data to better understand the experiences of direct and vicarious COVID-19-related discrimination or racism. Gathering quantitative and qualitative data on COVID-19-related racism will provide a better understanding of the recent experiences of racial and ethnic groups, identify specific racial and ethnic groups that may be more susceptible to racism, and inform strategies that are tailored to preventing stigma among specific racial and ethnic groups in the event of another pandemic.

Given these knowledge gaps, this study aimed to: (1) determine the prevalence of direct and vicarious COVID-19-related racism, (2) examine whether experiencing direct and vicarious COVID-19-related racism varied across racial and ethnic groups, (3) identify themes in open-ended responses elaborating on direct and vicarious experiences of COVID-19-related racism, and (4) examine the prevalence of themes in the overall population and across racial and ethnic groups in the U.S.

## Methods

### Data Source

Data were obtained from the Race-Related Experiences Associated with COVID-19 and Health in the United States (REACH-US) study, which was an online nationally representative survey that was administered in English and Spanish (Hispanic/Latino population only) by YouGov, Inc to adults (≥ 18 years old) living in the U.S. between January 26, 2021 and March 3, 2021. The REACH-US study included 1000 White, 1000 Black/African American, 1000 Hispanic/Latino, 1000 Asian, 500 American Indian/Alaska Native, 500 Native Hawaiian/Pacific Islander, and 500 Multiracial adults. Eligible participants were recruited from the YouGov proprietary research panel (~ 1.8 million adults). Participants were then matched to a theoretical target sample generated from the 2018 American Community Survey using demographic characteristics. After matching, propensity scores generated through logistic regression, adjusting for age, gender, years of education, and region, were used to generate sampling weights. This combination of matching and weighting resulted in a nationally representative sample within each racial and ethnic group. Additional details about the REACH-US study have been described elsewhere [16]. Given that YouGov provided de-identified data to the research team, this study was determined to be exempt, non-human subjects research by the Institutional Review Board at the National Institutes of Health. The data are available from the corresponding author upon reasonable request.

For this analysis, 5,476 participants who had complete data on all study variables of interest were included in the quantitative analyses (*N* = 5,480 weighted). Twenty-four participants were excluded due to missing data on age, gender, annual household income, or vicarious COVID-19-related racism. Participants who reported experiencing direct or vicarious COVID-19-related racism experiences and provided open-ended responses to the follow-up questions were included in the qualitative analyses (direct, *n* = 368; vicarious, *n* = 947). One participant who experienced direct COVID-19-related racism did not provide a response to the follow-up question and was therefore excluded from the qualitative analyses.

### Measures

#### Direct and Vicarious COVID-19-Related Racism.

COVID-19-related racism was captured using two single-item questions. Direct COVID-19-related racism was measured with the question: “Since the start of the pandemic, have you been the target of racism because you were thought to belong to a group that is more likely to get COVID-19?” (No; Yes). Vicarious COVID-19-related racism was measured with the question: “Since the start of the pandemic, have you witnessed any incidents of racism against persons who were thought to belong to a group that is more likely to get COVID-19?” (No; Yes).

Participants who reported experiencing direct COVID-19-related racism were asked to elaborate on their experiences of being the target of racism because they were thought to belong to a group that is more likely to get COVID-19. A similar open-ended item was provided to participants who reported vicarious COVID-19-related racism and they were asked to elaborate on their experiences of witnessing any incidents of racism against persons who were thought to belong to a group that is more likely to get COVID-19.

#### Race and Ethnicity.

Participants self-identified their race and ethnicity as either White, Black/African American, Hispanic/Latino, Asian, American Indian/Alaska Native, Native Hawaiian/Pacific Islander, or Multiracial. Participants who self-identified as Hispanic/Latino were also asked “In which language do you prefer to take the survey?”. Based on language preference, the Hispanic/Latino participants were grouped into Hispanic/Latino English Language Preference (ELP) and Hispanic/Latino Spanish Language Preference (SLP).

#### Covariates.

Age in years (18–34; 35–49; 50–64; and ≥ 65), gender (man; woman; and combined non-binary, transgender, not listed-given the small sample sizes), highest educational attainment (≤ high school diploma or equivalent diploma; some vocational, trade school, some college or associate degree; Bachelor’s degree; and graduate or professional degree), and annual household income (< $20,000; $20,000-$49,999; $50,000-$99,999; and ≥ $100,000) were included in the analyses as covariates. These covariates were selected based on previous literature and treated as confounding variables, which is consistent with much of the literature.

### Data Analyses

#### Quantitative Analyses.

All analyses were conducted using SAS version 9.4 (SAS Inc, Cary, NC) and weighted to produce nationally representative estimates within each racial and ethnic group. Descriptive analyses were conducted to describe the population characteristics and the prevalence of COVID-19-related racism overall and across racial and ethnic groups. Binomial logistic regression, adjusting for age, gender, highest educational attainment, and annual household income was used to estimate the odds of experiencing direct and vicarious COVID-19-related racism, respectively, across racial and ethnic groups. In addition, secondary analyses were performed to assess whether a four-level measure of COVID-19-related racism (i.e., both direct and vicarious, direct only, vicarious only, no experiences [reference category]) would yield similar results as those in the initial analyses that measured direct and vicarious COVID-19-related racism separately. Multinomial logistic regression, adjusting for the abovementioned covariates, estimated the associations between race and ethnicity and the 4-level measure of COVID-19-related racism.

#### Qualitative Analyses.

A qualitative approach using inductive thematic analyses was conducted. First, two researchers (YJC, JSS) reviewed the two open-ended responses describing direct and vicarious experiences of COVID-19-related racism. Based on this review, the two reviewers developed an initial set of codes to capture common descriptions of direct and vicarious COVID-19-related racism in the open-ended responses. Next, the entire review team (YJC, JSS, JRF, ATF) developed the codebook of themes from the initial codes. Given that similar codes emerged from the descriptions of direct and vicarious COVID-19-related racism, the same set of themes was developed for both direct and vicarious COVID-19-related racism and included in the codebook. Following this step, two researchers (YJC, JSS) independently reviewed all open-ended responses and assigned themes to each response. More than one theme or subtheme were assigned to each response if the response addressed multiple topics. Next, the two reviewers (YJC, JSS) determined whether their separate assignment of the themes to responses was consistent, and when appropriate, any discrepancies in the coding were reconciled by the entire review team (YJC, JSS, JRF, ATF).

Themes assigned to less than 1% of responses across both direct and vicarious COVID-19-related racism were deleted from the list of themes and were excluded from the final codebook. An additional 59 responses for direct COVID-19-related racism and 86 responses for vicarious COVID-19-related racism were coded as irrelevant, unclear, and insufficient.

## Results

### Quantitative Findings

#### Population Characteristics.

Characteristics of the total study population and within each racial and ethnic group (weighted) are described in Table 1 (Table A.1 includes unweighted characteristics). In the overall study population, participants were 44.3 years of age on average, mostly women (54.3%), attained post high school education (60.2%), and had an annual household income of at least $20,000 (70.3%). There were significant differences in sociodemographic characteristics across racial and ethnic groups (*p-*value < 0.05) (Table 1).

**Table 1:** Distribution of participant characteristics in the REACH-US study (weighted)

	Overall (N = 5480)	American Indian/ Alaska Native (n = 499)	Asian (n = 998)	Black/ African American (n = 997)	Hispanic/ Latino (ELP)^a^ (*n* = 495)	Hispanic/ Latino (SLP)^b^ (*n* = 504)	Multiracial (*n* = 497)	Native Hawaiian/ Pacific Islander (*n* = 500)	White (*n* = 990)
Age in years, mean (SD)	44.3 (0.3)	44.7 (1.0)	44.2 (0.8)	45.0 (0.6)	41.8 (0.9)	40.9 (0.7)	39.7 (0.9)	41.4 (0.9)	50.2 (0.6)
Age in years, n (%)									
18–34	1905 (34.8)	177 (35.4)	339 (34.0)	332 (33.3)	209 (42.3)	179 (35.5)	235 (47.3)	184 (36.8)	250 (25.3)
35–49	1469 (26.8)	114 (22.9)	295 (29.5)	239 (24.0)	133 (26.7)	183 (36.4)	124 (24.9)	171 (34.3)	210 (21.2)
50–64	1301 (23.7)	130 (26.1)	230 (23.1)	268 (26.9)	66 (13.4)	124 (24.5)	88 (17.7)	110 (22.0)	285 (28.8)
65 +	805 (14.7)	78 (15.6)	134 (13.4)	158 (15.8)	87 (17.6)	18 (3.6)	50 (10.1)	35 (6.9)	245 (24.7)
Gender, n (%)									
Man	2405 (43.9)	176 (35.2)	453 (45.4)	463 (46.4)	244 (49.2)	194 (38.5)	229 (46.1)	168 (33.7)	477 (48.2)
Woman	2978 (54.3)	311 (62.2)	531 (53.2)	527 (52.9)	246 (49.7)	308 (61.1)	236 (47.4)	317 (63.3)	503 (50.8)
Non-binary	59 (1.1)	11 (2.3)	6 (0.6)	6 (0.6)	3 (0.6)	1 (0.2)	22 (4.4)	2 (0.3)	9 (0.9)
Transgender	19 (0.3)	1 (0.3)	2 (0.2)	1 (0.1)	1 (0.2)	1 (0.2)	1 (0.3)	11 (2.2)	0
Gender not listed	19 (0.4)	0	6 (0.6)	0	1 (0.3)	0	9 (1.8)	2 (0.5)	1 (0.1)
Highest educational attainment, n (%)									
≤ High school	2183 (39.8)	212 (42.4)	236 (23.7)	422 (42.4)	263 (53.1)	338 (67.1)	160 (32.2)	229 (45.8)	322 (32.6)
Some college^c^	1806 (33.0)	222 (44.5)	238 (23.8)	379 (38.0)	154 (31.1)	106 (21.0)	189 (37.9)	189 (37.9)	330 (33.3)
Bachelor’s degree	912 (16.6)	40 (8.1)	303 (30.4)	119 (12.0)	43 (8.8)	46 (9.2)	93 (18.8)	58 (11.6)	208 (21.0)
Graduate/ professional	579 (10.6)	25 (5.0)	221 (22.1)	77 (7.6)	35 (7.0)	14 (2.7)	55 (11.1)	24 (4.7)	130 (13.1)
Annual household income, n (%)									
< $20,000	1627 (29.7)	214 (42.8)	182 (18.2)	412 (41.3)	145 (29.4)	161 (32.0)	123 (24.8)	189 (37.9)	200 (20.2)
$20,000–49,999	1694 (30.9)	149 (29.8)	257 (25.7)	301 (30.2)	176 (35.5)	246 (48.8)	138 (27.7)	133 (26.6)	295 (29.8)
$50,000–99,999	1375 (25.1)	100 (20.1)	286 (28.7)	194 (19.5)	123 (24.8)	77 (15.3)	164 (33.1)	130 (26.0)	301 (30.4)
≥ $100,000	784 (14.3)	36 (7.3)	273 (27.4)	90 (9.0)	51 (10.3)	20 (3.9)	72 (14.4)	48 (9.5)	194 (19.6)

There were statistically significant differences for all population characteristics across racial and ethnic groups (p-values < 0.05)

^a^ELP = English Language Preference

^b^SLP = Spanish Language Preference

^c^Includes trade school and associate degree

#### Prevalence of COVID-19-Related Racism.

**Fig. 1:**
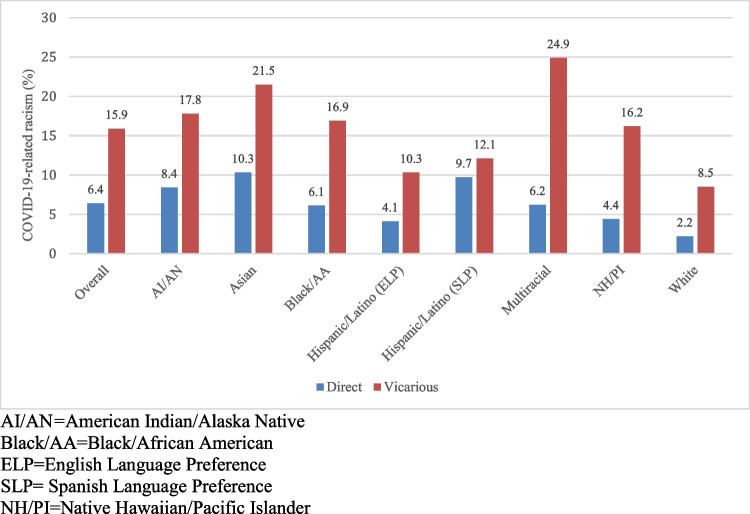
Prevalence of direct and vicarious COVID-19-related racism across racial and ethnic groups (weighted)

Overall, 6.4% of adults reported experiencing direct COVID-19-related racism and 15.9% reported experiencing vicarious COVID-19-related racism (Fig. 1). Asian adults (10.3%) reported the highest prevalence of direct COVID-19-related racism, followed by Hispanic/Latino (SLP) (9.7%), American Indian/Alaska Native (8.4%), Multiracial (6.2%), Black/African American (6.1%), Native Hawaiian/Pacific Islander (4.4%), Hispanic/Latino (ELP) (4.1%), and White (2.2%) adults. Multiracial adults (24.9%) reported the highest prevalence of vicarious COVID-19-related racism, followed by Asian (21.5%), American Indian/Alaska Native (17.8%), Black/African American (16.9%), Native Hawaiian/Pacific Islander (16.2%), Hispanic/Latino (SLP) (12.1%), Hispanic/Latino (ELP) (10.3%), and White (8.5%) adults.

#### Odds of Experiencing COVID-19-Related Racism Across Racial and Ethnic Groups.

Compared to White adults, all other racial and ethnic groups (except for Hispanic/Latino (ELP)) had significantly higher odds of experiencing direct COVID-19-related racism (AORs: 2.06–4.92), with Asian (AOR: 4.92 [95% CI: 2.84–8.50]), Hispanic/Latino (SLP) (AOR: 4.74 [95% CI: 2.58–8.72]), and American Indian/Alaska Native (AOR: 4.08 [95% CI: 2.11–7.92]) having the highest odds. Vicarious COVID-19-related racism was also significantly higher among all racial and ethnic groups except for Hispanic/Latino (ELP) (AORs: 1.63–3.02), with Multiracial (AOR: 3.02 [95% CI: 2.18–4.18]), Asian (AOR: 2.54 [95% CI: 1.88–3.42]), and American Indian/Alaska Native (AOR: 2.38 [95% CI: 1.59–3.56]) having the highest odds (Table 2).

**Table 2:** Odds of experiencing direct and vicarious COVID-19-related racism (in two separate models) across racial and ethnic groups (weighted)

	Direct	Vicarious
	AOR^c^ (95% CI)^d^	AOR^c^ (95% CI)^d^
Race and Ethnicity		
American Indian/Alaska Native	4.08 (2.11, 7.92)	2.38 (1.59, 3.56)
Asian	4.92 (2.84, 8.50)	2.54 (1.88, 3.42)
Black/African American	2.81 (1.57, 5.01)	2.31 (1.72, 3.09)
Hispanic/Latino (ELP)^a^	1.86 (0.91, 3.77)	1.25 (0.84, 1.86)
Hispanic/Latino (SLP)^b^	4.74 (2.58, 8.72)	1.63 (1.10, 2.42)
Multiracial	2.75 (1.39, 5.42)	3.02 (2.18, 4.18)
Native Hawaiian/Pacific Islander	2.06 (1.06, 4.01)	2.01 (1.39, 2.90)
White (reference)	1.00 (reference)	1.00 (reference)

^a^ELP = English Language Preference

^b^SLP = Spanish Language Preference

^c^AOR = Adjusted Odds Ratio

^d^Model adjusted for age, gender, highest educational attainment, and annual household income

Secondary analysis indicated that compared to White adults, all other racial and ethnic groups had significantly higher odds of experiencing both direct and vicarious COVID-19-related racism experiences (AORs: 4.27–14.03), including Hispanic/Latino (ELP) (Table A.2). Compared to White adults, only Hispanic/Latino (SLP) adults had a significantly higher odds of experiencing only direct COVID-19-related racism (AOR: 2.86 [95% CI: 1.30–6.28]) than experiencing no COVID-19-related racism. However, American Indian/Alaska Native, Asian, Black/African American, Multiracial, and Native Hawaiian/Pacific Islander adults had a significantly higher odds of experiencing only vicarious COVID-19-related racism (AORs: 1.66–2.48) than experiencing no COVID-19-related racism. Similar to the findings for any direct and any vicarious COVID-19-related racism, compared to White adults, all other racial and ethnic groups had significantly higher odds of simultaneously experiencing direct and vicarious COVID-19-related racism. However, compared to findings from the main analyses, findings from secondary analyses indicated that fewer racial and ethnic groups had a significantly higher odds of experiencing only direct (the Hispanic/Latino (SLP) group) or only vicarious COVID-19-related racism (American Indian/Alaska Native, Asian, Black/African American, Multiracial, and Native Hawaiian/Pacific Islander groups).

### Qualitative Findings

Table A.3 includes the codebook with descriptions of themes, subthemes, and illustrated quotes of direct and vicarious experiences of COVID-19-related racism. Open-ended responses describing experiences of direct (*n* = 368) COVID-19-related racism (Table 3) and vicarious (*n* = 947) COVID-19-related racism (Table 4) were grouped into two domains – *type of mistreatment* and *settings where racism occurred*.

**Table 3:** Open-ended responses describing experiences of direct COVID-19-related racism across racial and ethnic groups (unweighted)

		Overall (n = 368)	American Indian/ Alaska Native (*n* = 41)	Asian (*n* = 119)	Black/ African American (*n* = 60)	Hispanic/ Latino (ELP)^a^ (*n* = 19)	Hispanic/ Latino (SLP)^b^ (*n* = 46)	Multiracial (*n* = 29)	Native Hawaiian/ Pacific Islander (*n* = 34)	White (*n* = 20)
Domain	Theme and Subtheme	***n*** (%)	***n*** (%)	***n*** (%)	***n*** (%)	***n*** (%)	***n*** (%)	***n*** (%)	***n*** (%)	***n*** (%)
Type of mistreatment	**Physical mistreatment**	10 (2.7)	1 (2.4)	4 (3.4)	3 (5.0)	0	0	1 (3.4)	0	1 (5.0)
	**Verbal mistreatment**	59 (16.0)	10 (24.4)	31 (26.1)	1 (1.7)	1 (5.3)	1 (2.2)	9 (31.0)	4 (11.8)	2 (10.0)
	**Covert mistreatment**	62 (16.8)	6 (14.6)	31 (26.1)	7 (11.7)	2 (10.5)	4 (8.7)	7 (24.1)	2 (5.9)	3 (15.0)
	**Police brutality**	6 (1.6)	0	0	3 (5.0)	0	0	2 (6.9)	1 (2.9)	0
	**Medical mistreatment/health disparity**	15 (4.1)	5 (12.2)	1 (0.8)	3 (5.0)	1 (5.3)	1 (2.2)	1 (3.4)	2 (5.9)	1 (5.0)
	**Mistreatment specific to COVID-19**	70 (19.0)	4 (9.8)	44 (37.0)	3 (5.0)	5 (26.3)	6 (13.0)	3 (10.3)	3 (8.8)	2 (10.0)
	High COVID-19 threat from being a member of a vulnerable group	7 (1.9)	0	0	1 (1.7)	1 (5.3)	2 (4.3)	0	1 (2.9)	2 (10.0)
	High COVID-19 threat from not engaging in COVID-19 preventive behaviors/actions	6 (1.6)	3 (7.3)	1 (0.8)	0	1 (5.3)	1 (2.2)	0	0	0
	High COVID-19 threat from beliefs about disease origin or the carrier	57 (15.5)	1 (2.4)	43 (36.1)	2 (3.3)	3 (15.8)	3 (6.5)	3 (10.3)	2 (5.9)	0
	**Mistreatment specific to race and ethnicity**	58 (15.8)	6 (14.6)	19 (16.0)	8 (13.3)	1 (5.3)	5 (10.9)	6 (20.7)	7 (20.6)	6 (30.0)
	Reference to racial and ethnic groups	49 (13.3)	4 (9.8)	19 (16.0)	7 (11.7)	1 (5.3)	5 (10.9)	4 (13.8)	7 (20.6)	2 (10.0)
	Black Lives Matter Movement	2 (0.5)	1 (2.4)	0	1 (1.7)	0	0	0	0	0
	Reverse racism	8 (2.2)	2 (4.9)	0	0	0	0	2 (6.9)	0	4 (20.0)
	**General mistreatment**	55 (14.9)	7 (17.1)	10 (8.4)	11 (18.3)	6 (31.6)	6 (13.0)	6 (20.7)	5 (14.7)	4 (20.0)
Settings where racism occurred	**Places of mistreatment**	80 (21.7)	8 (19.5)	33 (27.7)	12 (20.0)	7 (36.8)	7 (15.2)	8 (27.6)	3 (8.8)	2 (10.0)
	Public/street	10 (2.7)	0	8 (6.7)	1 (1.7)	0	0	1 (3.4)	0	0
	Medical facility	15 (4.1)	4 (9.8)	1 (0.8)	1 (1.7)	2 (10.5)	1 (2.2)	3 (10.3)	2 (5.9)	1 (5.0)
	Work/school	13 (3.5)	0	3 (2.5)	4 (6.7)	2 (10.5)	0	3 (10.3)	0	1 (5.0)
	Asian business	0 (0)	0	0	0	0	0	0	0	0
	Restaurants/stores	31 (8.4)	3 (7.3)	15 (12.6)	3 (5.0)	3 (15.8)	5 (10.9)	1 (3.4)	1 (2.9)	0
	Government	5 (1.4)	0	3 (2.5)	2 (3.3)	0	0	0	0	0
	Other settings	10 (2.7)	1 (2.4)	6 (5.0)	1 (1.7)	0	1 (2.2)	1 (3.4)	0	0
	**Mistreatment in media**	12 (3.3)	1 (2.4)	3 (2.5)	3 (5.0)	1 (5.3)	0	4 (13.8)	0	0
	**Mistreatment related to politics**	12 (3.3)	2 (4.9)	8 (6.7)	0	0	0	2 (6.9)	0	0

^a^ELP = English Language Preference

^b^SLP = Spanish Language Preference

^*^Given that the categories were not mutually exclusive, and responses could belong to multiple themes, percentages did not add to 100%

**Table 4:** Open-ended responses describing experiences of vicarious COVID-19-related racism across racial and ethnic groups (unweighted)

		Overall (*n* = 947)	American Indian/ Alaska Native (*n* = 83)	Asian (*n* = 241)	Black/ African American (*n* = 172)	Hispanic/ Latino (ELP)^a^ (*n* = 56)	Hispanic/ Latino (SLP)^b^ (*n* = 57)	Multiracial (*n* = 131)	Native Hawaiian/ Pacific Islander (*n* = 102)	White (*n* = 105)
Domain	Theme and Subtheme	***n*** (%)	***n*** (%)	***n*** (%)	***n*** (%)	***n*** (%)	***n*** (%)	***n*** (%)	***n***(%)	***n***(%)
Type of mistreatment	**Physical mistreatment**	39 (4.1)	3 (3.6)	15 (6.2)	3 (1.7)	0	0	11 (8.4)	3 (2.9)	4 (3.8)
	**Verbal mistreatment**	110 (11.6)	4 (4.8)	49 (20.3)	9 (5.2)	3 (5.4)	3 (5.3)	21 (16.0)	5 (4.9)	16 (15.2)
	**Covert mistreatment**	84 (8.9)	6 (7.2)	23 (9.5)	17 (9.9)	4 (7.1)	5 (8.8)	10 (7.6)	10 (9.8)	9 (8.6)
	**Police brutality**	54 (5.7)	3 (3.6)	6 (2.5)	13 (7.6)	5 (8.9)	0	9 (6.9)	4 (3.9)	14 (13.3)
	**Medical mistreatment/health disparity**	105 (11.1)	15 (18.1)	9 (3.7)	40 (23.3)	7(12.5)	4 (7.0)	12 (9.2)	5 (4.9)	13 (12.4)
	**Mistreatment specific to COVID-19**	197 (20.8)	8 (9.6)	75 (31.1)	26 (15.1)	6 (10.7)	7 (12.3)	32 (24.4)	19 (18.6)	31 (29.5)
	High COVID-19 threat from being a member of a vulnerable group	16 (1.7)	1 (1.2)	1 (0.4)	5 (2.9)	0	0	1 (0.8)	4 (3.9)	4 (3.8)
	High COVID-19 threat from not engaging in COVID-19 preventive behaviors/actions	23 (2.4)	2 (2.4)	3 (1.2)	5 (2.9)	2 (3.6)	2 (3.5)	3 (2.3)	2 (2.0)	4 (3.8)
	High COVID-19 threat from beliefs about disease origin or the carrier	166 (17.5)	5 (6.0)	71 (29.5)	17(9.9)	4 (7.1)	5 (8.8)	28 (21.4)	13 (12.7)	23 (21.9)
	**Mistreatment specific to race and ethnicity**	120 (12.7)	11 (13.3)	26 (10.8)	17 (9.9)	10 (17.9)	5 (8.8)	18 (13.7)	17 (16.7)	16 (15.2)
	Reference to racial and ethnic groups	81 (8.6)	7 (8.4)	23 (9.5)	13 (7.6)	6 (10.7)	5 (8.8)	11 (8.4)	12 (11.8)	4 (3.8)
	Black Lives Matter Movement	33 (3.5)	4 (4.8)	2 (0.8)	4 (2.3)	3 (5.4)	0	5 (3.8)	4 (3.9)	11 (10.5)
	Reverse racism	8 (0.8)	0	1 (0.4)	0	2 (3.6)	0	2 (1.5)	1 (0.9)	2 (1.9)
	**General mistreatment**	205 (21.6)	18 (21.7)	56 (23.2)	31 (18.0)	17 (30.4)	10 (17.5)	31 (23.7)	27 (26.5)	15 (14.3)
Settings where racism occurred	**Places of mistreatment**	180 (19. 0)	9 (10.8)	48 (19.9)	47 (27.3)	8 (14.3)	10 (17.5)	27 (20.6)	15 (14.7)	16 (15.2)
	Public/street	20 (2.1)	1 (1.2)	11 (4.6)	1 (0.6)	2 (3.6)	1 (1.8)	3 (2.3)	0	1 (1.0)
	Medical facility	49 (5.2)	5 (6.0)	4 (1.7)	21 (12.2)	1 (1.8)	2 (3.5)	10 (7.6)	3 (2.9)	3 (2.9)
	Work/school	43 (4.5)	5 (6.0)	9 (3.7)	9 (5.2)	3 (5.4)	4 (7.0)	6 (4.6)	4 (3.9)	8 (7.6)
	Asian business	16 (1.7)	0	5 (2.1)	2 (1.2)	0	0	6 (4.6)	1 (1.0)	2 (1.9)
	Restaurants/stores	37 (3.9)	2 (2.4)	11 (4.6)	8 (4.7)	2 (3.6)	3 (5.3)	5 (3.8)	5 (4.9)	1 (1.0)
	Government	13 (1.4)	0	4 (1.7)	6 (3.5)	1 (1.8)	0	1 (0.8)	0	1 (1.0)
	Other settings	17 (1.8)	1 (1.2)	6 (2.5)	2 (1.2)	0	1 (1.8)	3 (2.3)	2 (2.0)	2(1.9)
	**Mistreatment in media**	144 (15.2)	12 (14.5)	42 (17.4)	24 (14.0)	5 (8.9)	2 (3.5)	28 (21.4)	9 (8.8)	22 (21.0)
	**Mistreatment related to politics**	66 (7.0)	4 (4.8)	11 (4.6)	15 (8.7)	3 (5.4)	0	14 (10.7)	5 (4.9)	14 (13.3)

^a^ELP = English Language Preference

^b^SLP = Spanish Language Preference

^*^Given that the categories were not mutually exclusive, and responses could belong to multiple themes, percentages did not add to 100%

#### Type of Mistreatment.

Eight themes capturing experiences of direct and vicarious types of mistreatment were identified: physical mistreatment, verbal mistreatment, covert mistreatment, police brutality, medical mistreatment/health disparity, mistreatment specific to COVID-19, mistreatment specific to race and ethnicity, and general mistreatment (Table A.3).

Among participants who experienced direct COVID-19-related racism (*n* = 368), the most reported *type of mistreatment* was *mistreatment specific to COVID-19* (19.0%), and specifically, *high COVID-19 threat from beliefs about disease origin or the carrier* (15.5%) (Table 3). Asian and Hispanic/Latino SLP adults most commonly reported *mistreatment specific to COVID-19* (37.0% and 13.0%, respectively), whereas Black/African American and Hispanic/Latino (both ELP and SLP) adults most frequently reported *general mistreatment* (18.3%, 31.6%, 13.0%, respectively), American Indian/Alaska Native and Multiracial adults most frequently reported *verbal mistreatment* (24.4% and 31.0%, respectively), and Native Hawaiian/Pacific Islander and White adults most commonly reported experiencing *mistreatment specific to race and ethnicity* (20.6% and 30.0%, respectively).

The most reported *type of mistreatment* identified by participants who experienced vicarious COVID-19-related racism (*n* = 947) was witnessing *general mistreatment* (21.6%) (Table 4). Witnessing *general mistreatment* was reported the most among American Indian/Alaska Native (21.7%), Hispanic/Latino (both ELP and SLP) (30.4% and 17.5%, respectively), and Native Hawaiian/Pacific Islander (26.5%) adults. Asian (31.1%), Multiracial (24.4%), and White (29.5%) adults most frequently reported witnessing *mistreatment specific to COVID-19*, whereas Black/African American adults most frequently reported *medical mistreatment/health disparity* (23.3%).

#### Settings Where Racism Occurred.

Across both direct and vicarious COVID-19-related racism experiences, three themes emerged within the domain of settings where racism occurred: places of mistreatment (e.g., restaurants/stores), mistreatment in media (e.g., news), and mistreatment related to politics (e.g., political leader referring to the COVID-19 virus as the ‘Wuhan virus’) (Table A.3).

Among participants who experienced direct COVID-19-related racism (Table 3), the most reported theme within the domain of *settings where racism occurred* was *places of mistreatment* (21.7%), particularly in *restaurants/stores* (8.4%). Although the most reported subtheme for those who experienced vicarious COVID-19-related racism was *medical facility* (5.2%) within the theme of *places of mistreatment*, the theme *mistreatment in media* (15.2%) was reported more frequently than *medical facility* (Table 4).

## Discussion

### Quantitative Findings

There have been several studies that focused on COVID-19-related discrimination or racism [1, 3, 6, 9–15], but fewer studies focused on both direct and vicarious types of COVID-19-related discrimination or racism [9, 10, 14, 15]. The present study expanded on these previous studies by using both quantitative and qualitative approaches to examine the prevalence of both direct and vicarious COVID-19-related racism across nationally representative racial and ethnic groups in the U.S.

Consistent with findings from previous studies [9, 10, 14, 15], participants in the present study reported experiencing more vicarious COVID-19-related racism (15.9%) than direct COVID-19-related racism (6.4%). Observing more vicarious than direct COVID-19-related racism could be due to the social isolation restrictions during the COVID-19 pandemic, which limited in-person interactions and contributed to an increase in media viewing during that time.

The prevalence estimates of direct COVID-19-related racism in the current study and in two previous studies (range: 4.0% to 10.0%) [6, 10], were relatively lower than the prevalence estimates (COVID-19-related discrimination or racism) observed in other studies (range: 22.1% to 58.7%) [3, 9, 11–15]. These differences in findings may be due to multiple factors such as differences in population characteristics (e.g., racial and ethnic groups examined), or the measures used to capture COVID-19-related discrimination or racism. The combination of these factors could have contributed to the higher prevalence estimates in other studies. For example, while some studies exclusively focused on Asian American individuals [9, 11, 12, 15], other studies, including the current one, examined differences in COVID-19-related racism across racial and ethnic groups [3, 6, 10, 13, 14]. In addition, direct COVID-19-related racism experiences in the current study and in two previous studies were measured by using single items [10, 12] whereas other studies assessed COVID-19-related discrimination or racism by using multiple items [3, 9, 11, 13–15].

A lower prevalence of vicarious COVID-19-related racism was observed in the current study (15.9%) than in other studies (range: 45.9% to 91.9%) [9, 10, 14, 15]. Similar to direct COVID-19-related racism, differences in the population characteristics (e.g., Asian race [9, 15], college student [10, 15], former or current tobacco user [14]), or the measures used to capture vicarious COVID-19-related discrimination or racism [9, 15] may also explain why the prevalence estimates in other studies were higher than those observed in the current study.

Our findings across racial and ethnic groups were consistent with findings from one prior study on direct COVID-19-related discrimination [3], whereby marginalized racial and ethnic groups (i.e., American Indian/Alaska Native, Asian, Black/African American, Hispanic/Latino (SLP), Multiracial, and Native Hawaiian/Pacific Islander groups) had higher odds of experiencing direct COVID-19-related racism compared to the White racial group. Although the Hispanic/Latino (ELP) group did not have significantly higher odds of experiencing direct COVID-19-related racism compared to the White racial group (i.e., the confidence interval included 1), the effect sizes were large and may have reached statistical significance with a larger sample size. Alternatively, these findings may suggest that there are distinct differences in the experiences of Hispanic/Latino ELP and Hispanic/Latino SLP participants. For example, language and skin color are key factors that inform the characterization and discrimination experiences of the Hispanic/Latino population in the U.S. Therefore, the Hispanic/Latino ELP participants in this study may have been viewed as non-Hispanic because they spoke English and/or may have phenotypically presented as White, which could have influenced their discrimination experiences [17]. However, the racial and ethnic group with the highest prevalence of direct discrimination or racism varied within and across previous studies, depending on the measure of direct COVID-19-related racism or the time of measurement. Consistent with findings from the present study, most studies [3, 10] found the highest prevalence of direct COVID-19-related racism or discrimination experiences among Asian adults than among other racial and ethnic groups. These findings could be explained by the anti-Asian rhetoric associated with COVID-19 and the increase in violence against Asian adults in the U.S. [2, 18].

The highest prevalence of vicarious COVID-19-related racism, on the other hand, was reported among Multiracial adults in the present study. Multiracial participants who have multiple racial identities could have been exposed to more vicarious discrimination because they may be more attuned to the experiences of others who share those same identities. Given that this study did not collect data on the separate racial categories that comprised the Multiracial group, this potential explanation for the highest prevalence of vicarious COVID-19-related racism among the Multiracial group could not be confirmed. However, two prior studies observed the highest prevalence of vicarious COVID-19-related discrimination or racism among Asian adults [10, 14]. These differences in the current study and two prior studies could be due to the lack of a separate Multiracial ethnic group in one prior study [14] or to differences in the racial and ethnic composition of the Multiracial ethnic group [10].

### Qualitative Findings

The two domains identified from the qualitative responses on direct and vicarious COVID-19-related racism in this study were *type of mistreatment* and *settings where racism occurred*. Previous studies also organized themes into types of discrimination or racism [5, 7, 19, 20]. In contrast to the current study, prior studies did not ask participants to separately elaborate on their experiences of direct and vicarious COVID-19-related discrimination or racism, yet many of the themes and subthemes in prior studies indicated direct and vicarious discrimination or racism experiences [7, 19–21]. Some themes and subthemes for direct and vicarious COVID-19-related discrimination or racism overlapped with those in previous studies, with some notable differences.

Given that this study specifically asked about experiences of racism that were deeply related to stigma around the COVID-19 pandemic, the theme *mistreatment specific to COVID-19* was endorsed across both direct and vicarious experiences, with the highest endorsement within the *type of mistreatment* domain for direct COVID-19-related racism experiences. However, in prior studies, the themes comprising direct discrimination or racism were based on interpersonal level experiences, which particularly reflect how direct discrimination or racism were manifested. Prior studies created themes or subthemes describing overt and/or covert acts of discrimination or racism that participants personally received [7, 19, 20]. The present study also identified similar themes, such as *physical mistreatment, verbal mistreatment,* and *covert mistreatment* within the *type of mistreatment* domain across both direct and vicarious experiences, yet those themes (i.e., *verbal mistreatment* and *covert mistreatment*) were more frequently observed for direct than vicarious COVID-19-related racism experiences. The *covert mistreatment* and* verbal mistreatment* themes had the second and third highest endorsement rates in direct COVID-19-related racism experiences within the *type of mistreatment* domain.

In contrast to the current study, which identified themes for direct racism within the domain *settings where racism occurred,* many prior studies did not identify the locations where direct discrimination occurred. In the present study, many participants who experienced direct COVID-19-related racism reported that racism mostly occurred in physical settings such as restaurants/stores. Given that most settings (except for essential places such as grocery stores and medical facilities) were restricted or avoided during the COVID-19 pandemic, *restaurants/stores* were where individuals could have encountered direct COVID-19-related racism.

The *general mistreatment* theme was endorsed the most for vicarious COVID-19-related racism experiences within the *type of mistreatment* domain, which could be due to difficulties identifying the specific type of mistreatment experienced by others. However, most themes for vicarious discrimination or racism in prior studies were based on indirect experiences of discrimination or racism that were linked to stigma associated with racial and ethnic background or culture [7, 20]. These themes created in prior studies reflect prevailing racism towards specific racial and ethnic groups, particularly towards Asian individuals, as the entire ethnic group was being blamed for the COVID-19 pandemic. Although this study included the subtheme *High COVID-19 threat from beliefs about disease origin or the carrier* that was greatly endorsed across both direct and vicarious COVID-19-related racism experiences, the theme appeared more often for vicarious than direct COVID-19-related racism experiences.

Consistent with findings from previous studies, where there were themes or subthemes that comprise experiences of vicarious discrimination or racism, particularly in political and media contexts [7, 19, 20], the present study revealed that *mistreatment in media* and *mistreatment related to politics* themes were endorsed more in vicarious than in direct COVID-19-related racism experiences. Viral video recordings of mistreatment across multiple media platforms, racial comments online or in the media, and political statements in the media could have contributed to increased exposure to vicarious racism. One prior study conducted among Asian American young adults during the COVID-19 pandemic reported vicarious racial discrimination that frequently occurred via media, hearing stories from close individuals, and witnessing others’ interactions [22].

### Limitations

Several limitations should be considered when interpreting these findings. First, given that the REACH-US study was an online survey, individuals with limited internet access were less likely to participate. This digital divide, especially for marginalized groups (e.g., specific racial and ethnic groups, socially disadvantaged groups) [23], could have resulted in an underestimation of the prevalence of COVID-19-related racism. Second, participants were only given the option to complete the survey in English or Spanish (Hispanic/Latino only), which also would have limited the ability for non-English or non-Spanish speaking individuals to participate and could have contributed to an underestimation of the prevalence of COVID-19-related racism. Third, the vicarious COVID-19-related racism measure that was used in this study referred to witnessing acts of racism committed against anyone regardless of the racial and ethnic group to which that person belonged, whereas the more common measure of vicarious racism refers specifically to witnessing or hearing racism from others who belong to the same racial and ethnic group [24]. Using the measure that was specific to one’s own race and ethnicity in the present study could have provided a more accurate account of vicarious COVID-19-related racism and highlighted additional racial and ethnic differences in these experiences. Finally, our cross-sectional survey was conducted between January and March 2021, and may not reflect the prevalence of COVID-19-related racism experienced or witnessed after this time.

### Strengths

This study had several strengths. The study included a large, nationally representative U.S. population of diverse racial and ethnic groups, which allowed for comparison of COVID-19-related racism experiences across American Indian/Alaska Native, Asian, Black/African American, Hispanic/Latino, Multiracial, Native Hawaiian/Pacific Islander, and White adults. In addition, the inclusion of Hispanic/Latino English Language Preference and Hispanic/Latino Spanish Language Preference groups allowed for the examination of differences in experiences of COVID-19-related racism by language preference. Additionally, incorporating two measures of COVID-19-related racism (both direct and vicarious COVID-19-related racism) specifically linked to stigma arising from the COVID-19 pandemic allowed for a more nuanced exploration of COVID-19-related racism experiences. In addition, the inclusion of open-ended responses elaborating on the experiences of direct and vicarious COVID-19-related racism was an additional method in which to better understand experiences of COVID-19-related racism across racial and ethnic groups, which otherwise would not have been captured using only quantitative responses.

## Conclusion

Findings from this study suggested that American Indian/Alaska Native, Asian, Black/African American, Hispanic/Latino (SLP), Multiracial, and Native Hawaiian/Pacific Islander adults were significantly more likely than White adults to experience both direct and vicarious COVID-19-related racism, which included many types of mistreatment occurring across various settings. Given that this study occurred relatively early in the COVID-19 pandemic, future studies should assess whether the prevalence of direct and vicarious experiences of COVID-19-related racism have changed over time, as well as the impact of COVID-19-related racism on adverse health outcomes. The use of quantitative and qualitative responses to examine both direct and vicarious experiences of COVID-19-related racism across several racial and ethnic groups in the present study provided a novel way to better understand COVID-19-related racism. Specifically, the qualitative findings from this study highlighted the need for public health interventions that are tailored to the specific COVID-19-related racism experiences of marginalized racial and ethnic groups to reduce and prevent COVID-19-related racism. Based on the qualitative findings where stigmatization often resulted from misconceptions about COVID-19 among marginalized racial and ethnic groups, future studies focusing on the impact of “anti-stigma campaigns” to reduce COVID-19-related racism [25] could contribute to meaningful interventions. Furthermore, given that the media was a common setting for vicarious COVID-19-related racism, “mass media interventions” [25] could be designed to prevent/reduce vicarious COVID-19-related racism.

## Supplementary Information

Below is the link to the electronic supplementary material.

Supplementary file1 (DOCX 34 KB)

## Declarations

### Disclaimers

The contents and views in this manuscript are those of the authors and should not be construed to represent the views of the National Institute on Minority Health and Health Disparities, National Institutes of Health, or the Department of Health and Human Services.
