# Supplementary material for: Direct and Vicarious Experiences of COVID-19-Related Racism Across Racial and Ethnic Groups in the United States
Source: J Racial Ethn Health Disparities. 2024 Nov 20;12(6):3587–600. doi: 10.1007/s40615-024-02159-x (PMC12644215; doi:10.1007/s40615-024-02159-x)
Supplement: Supplementary file 1 — Supplementary file1 (DOCX 34 KB) [file 40615_2024_2159_MOESM1_ESM.docx]

**Direct and Vicarious Experiences of COVID-19-Related Racism Across Racial/Ethnic Groups in the U.S.**

**Appendix**

**Table A.1. Distribution of Participant Characteristics in the REACH-US Study (Unweighted)**

|  | **Overall**  **(N=5476)** | **American Indian/ Alaska Native**  **(n= 499)** | **Asian**  **(n= 998)** | **Black/**  **African American**  **(n= 995)** | **Hispanic/ Latino (ELP)^1^**  **(n= 499)** | **Hispanic/ Latino (SLP)^2^**  **(n= 499)** | **Multiracial**  **(n= 497)** | **Native Hawaiian/ Pacific Islander**  **(n= 499)** | **White**  **(n=990)** |
| --- | --- | --- | --- | --- | --- | --- | --- | --- | --- |
| **Age in years, n (%)** |  |  |  |  |  |  |  |  |  |
| 18-34 | 1914 (34.9) | 160 (32.1) | 364 (36.5) | 294 (29.6) | 194 (38.9) | 171 (34.3) | 231 (46.5) | 249 (49.9) | 251 (25.4) |
| 35-49 | 1515 (27.7) | 120 (24.0) | 316 (31.7) | 248 (24.9) | 151 (30.3) | 200 (40.1) | 133 (26.7) | 144 (28.9) | 203 (20.5) |
| 50-64 | 1187 (21.7) | 136 (27.3) | 212 (21.2) | 274 (27.5) | 68 (13.6) | 107 (21.4) | 75 (15.1) | 75 (15.0) | 240 (24.2) |
| 65+ | 860 (15.7) | 83 (16.6) | 106 (10.6) | 179 (18.0) | 86 (17.2) | 21 (4.2) | 58(11.7) | 31 (6.2) | 296 (29.9) |
| **Gender, n (%)** |  |  |  |  |  |  |  |  |  |
| Man | 2319 (42.3) | 186 (37.3) | 473 (47.4) | 426 (42.8) | 216 (43.3) | 174 (34.9) | 205 (41.3) | 161 (32.3) | 478 (48.3) |
| Woman | 3060 (55.9) | 303 (60.7) | 511 (51.2) | 563 (56.6) | 275 (55.1) | 323 (64.7) | 260 (52.3) | 324 (64.9) | 501 (50.6) |
| Non-binary | 61 (1.1) | 9 (1.8) | 8 (0.8) | 5 (0.5) | 3 (0.6) | 1 (0.2) | 22 (4.4) | 4 (0.8) | 9 (0.9) |
| Transgender | 15 (0.3) | 1(0.2) | 2 (0.2) | 1 (0.1) | 2 (0.4) | 1 (0.2) | 1 (0.2) | 7 (1.4) | 0 |
| Gender not listed | 21 (0.4) | 0 | 4 (0.4) | 0 | 3 (0.6) | 0 | 9 (1.8) | 3 (0.6) | 2 (0.2) |
| **Highest educational attainment, n (%)** |  |  |  |  |  |  |  |  |  |
| ≤ High school diploma | 1724 (31.5) | 155 (31.1) | 99 (9.9) | 368 (37.0) | 237 (47.5) | 318 (63.7) | 107 (21.5) | 159 (31.9) | 281 (28.4) |
| Some college/trade school/associate degree | 1907 (34.8) | 239 (47.9) | 263 (26.3) | 415 (41.7) | 140 (28.1) | 100 (20.1) | 204 (41.1) | 214 (42.9) | 332 (33.5) |
| Bachelor’s degree | 1096 (20.0) | 63 (12.6) | 365 (36.6) | 128 (12.9) | 52 (10.4) | 55 (11.0) | 121 (24.3) | 79 (15.8) | 233 (23.5) |
| Graduate/professional | 749 (13.7) | 42 (8.4) | 271 (27.2) | 84 (8.4) | 70 (14.0) | 26 (5.2) | 65 (13.1) | 47 (9.4) | 144 (14.6) |
| **Annual household income,**  **n (%)** |  |  |  |  |  |  |  |  |  |
| <$20,000 | 1505 (27.5) | 175 (35.1) | 147 (14.7) | 387 (38.9) | 145 (29.1) | 162 (32.5) | 123 (24.7) | 168 (33.7) | 198 (20.0) |
| $20,000-49,999 | 1654 (30.2) | 151 (30.3) | 235 (23.5) | 309 (31.1) | 166 (33.3) | 237 (47.5) | 132 (26.6) | 132 (26.5) | 292 (29.5) |
| $50,000-99,999 | 1418 (25.9) | 127 (25.4) | 291 (29.2) | 209 (21.0) | 125 (25.0) | 78 (15.6) | 162 (32.6) | 131 (26.2) | 295 (29.8) |
| ≥ $100,000 | 899 (16.4) | 46 (9.2) | 325 (32.6) | 90 (9.0) | 63 (12.6) | 22 (4.4) | 80 (16.1) | 68 (13.6) | 205 (20.7) |

There were statistically significant differences in population characteristics across racial/ethnic groups (p-value <0.05)

^1^ELP=English Language Preference

^2^SLP=Spanish Language Preference

**Table A.2. Odds of experiencing ‘only direct’, ‘only vicarious’, or ‘both direct and vicarious’ COVID-19-related racism versus none (in one model) across racial/ethnic groups (Weighted)**

|  | **Both direct and vicarious** | **Only direct** | **Only vicarious** |
| --- | --- | --- | --- |
|  | **AOR^3^ (95%CI)^4^** | **AOR^3^ (95%CI)^4^** | **AOR^3^ (95%CI)^4^** |
| **Race/Ethnicity** |  |  |  |
| American Indian/Alaska Native | 9.85 (3.51, 27.64) | 2.38 (0.99, 5.74) | 1.87 (1.19, 2.93) |
| Asian | 14.03 (5.70, 34.53) | 1.99 (0.95, 4.18) | 1.75 (1.24, 2.46) |
| Black/African American | 6.78 (2.66, 17.29) | 1.67 (0.78, 3.58) | 2.00 (1.46, 2.75) |
| Hispanic/Latino (ELP)^1^ | 4.27 (1.44, 12.69) | 0.98 (0.38, 2.55) | 1.02 (0.67, 1.57) |
| Hispanic/Latino (SLP)^2^ | 10.00 (3.76, 26.64) | 2.86 (1.30, 6.28) | 1.01 (0.62, 1.63) |
| Multiracial | 9.66 (3.53, 26.46) | 0.69 (0.24, 1.99) | 2.48 (1.76, 3.50) |
| Native Hawaiian/Pacific Islander | 6.35 (2.35, 17.15) | 0.61 (0.20, 1.86) | 1.66 (1.10, 2.49) |
| White (reference) | 1.00 (reference) | 1.00 (reference) | 1.00 (reference) |

^1^ELP=English Language Preference

^2^SLP=Spanish Language Preference

^3^AOR=Adjusted Odds Ratios

^4^Model was adjusted for age, gender, highest educational attainment, and annual household income

**Table A.3. Codebook**

| **Domain** | **Theme and Subtheme** | **Description** | **Illustrative quotes** | **Notes** |
| --- | --- | --- | --- | --- |
| **Type of mistreatment** | **Physical mistreatment** | When physical actions are intentionally used to harm another person (e.g., being slapped, pinched/punched, pushed, beaten, being intentionally coughed (spat) on, a victim of violence) | “There were cases where Asians were beaten because they were thought to be the carrier of the disease”  “White male in airport started intentionally coughing on me” |  |
|  | **Verbal mistreatment** | When the person or group is being verbally mistreated (e.g., derogatory language used to describe a racial group, name calling) | “Yes. My son experienced being called a Chinese virus in school for looking very Asian” ”I’ve had anti-Asian slurs hurled at me on the streets” |  |
|  | **Covert mistreatment** | When the mistreatment is in a subtle or an indirect form (e.g., avoided, followed, laughed at, received rude gestures or looks) | “A white older woman looked at me in fear when she got off the elevator as I was leaving the doctor’s office” “People were avoidant of international students from Asia” |  |
|  | **Police brutality** | When the mistreatment is related to acts by the police (e.g., the case of “George Floyd”) | “Black lives getting killed by police”  “George Floyd”  “Unable to sit inside of restaurants without being disturbed by police” | *The theme “police brutality” refers to acts by the police that involve any physical or verbal mistreatment. Therefore, responses coded as “police brutality” were not double coded as “physical mistreatment” or “verbal mistreatment” |
|  | **Medical mistreatment/health disparity** | When the mistreatment is from healthcare providers or is related to healthcare system disparities (e.g., uneven distribution of vaccine) | “Susan Moore” “Lack of access to health care for minorities” ”By the treatment or when I question about vaccine availability” | *Responses that refer to “the doctor who died” or “…a black doctor with COVID…” were assumed to be about Dr. Susan Moore who died in a medical facility, due to medical mistreatment. Therefore, those responses were double coded as "places of mistreatment (e.g., medical facility)” and “medical mistreatment/health disparity”) |
|  | **Mistreatment specific to COVID-19** |  |  |  |
|  | High COVID-19 threat from being a member of a vulnerable group | When the mistreatment is against people with certain occupations (e.g., healthcare workers, frontline/essential workers) who are at elevated risk of exposure to COVID-19 or are perceived by others to have increased exposure to COVID-19 | “Nurses and medical professional were viewed as a public danger” “I’m an essential worker and people accuse me of having COVID anytime I sniffle” |  |
|  | High COVID-19 threat from not engaging in COVID-19 preventive behaviors/actions | When the mistreatment is related to COVID-19 preventive behaviors/actions (e.g., social distancing, masking) | “Unmasked police rallies” “For not wearing a mask in a deserted street walking my dog” |  |
|  | High COVID-19 threat from beliefs about disease origin or the carrier | When the mistreatment is related to beliefs about the origin of COVID-19 or persons more likely to have COVID-19 (e.g., COVID-19 originated from Asian people or specific racial groups have COVID-19) | “Blacks been thrown out because it’s said that they carry the virus”  “Asian” people verbally blamed for the “China Virus”  “I have been blamed for COVID” | *Mistreatment directed at Chinese/Asian individuals but unclear whether mistreatment is caused by beliefs about COVID-19 (e.g., “The blatant racism against Chinese people”) should not be coded with this theme |
|  | **Mistreatment specific to race/ethnicity** |  |  |  |
|  | Reference to racial/ethnic groups | When responses mention mistreatment of specific racial/ethnic groups without providing additional information on mistreatment | “The Chinese” “White” “I am Black” “Latino” |  |
|  | Black Lives Matter Movement | When responses mention the Black Lives Matter movement | “The treatment of BLM protestors”  “Black Lives Matter” | *Responses that mention the U.S. National “capitol riot” should be coded with “mistreatment related to politics” and “places of mistreatment (e.g., “government”)”  *Responses like “Protests” should be included in “general mistreatment” since it is not specifically about the Black Lives Matter Movement |
|  | Reverse racism | When responses mention perceiving greater privilege among marginalized racial/ethnic groups compared to the White group | “White people are told to die by Black Lives Matter rioters” “I was because I have white privilege which is a lie” |  |
|  | **General mistreatment** | When responses include mistreatment, but the form of mistreatment is not clear enough to tell whether they are physical or verbal mistreatment (e.g., attacked, harassed, assaulted)  When responses include racism that refers to the definition of racism (“racism”, “targeted”, “treated differently”)  Or when mistreatment or the act occurred for reasons other than racism, responses should be considered as general mistreatment (e.g., homeless, underprivileged) | “News, Asian Americans being attacked” “Underprivileged”  “Yes, at the DMV by a member of another minority group- blacks” |  |
| **Settings where racism occurred** | **Places of mistreatment**  (e.g., public/street, medical facility, work/school, Asian business, restaurants/stores, government, and other setting) | Where mistreatment occurred | “My mother was treated with disrespect at a doctor’s office. She is Japanese”  “Called n****r, where, followed in stores by white security as we shopped, etc.” |  |
|  | **Mistreatment in media** | When the mistreatment is viewed or occurred in the media (e.g., on the internet, TV news, reports) | “Not in person, but I’ve seen plenty of videos of AAPIs getting harassed in grocery stores” “I have been told in group chats that I have COVID” | *Responses should specifically mention words like “online”, “TV”, or “report” |
|  | **Mistreatment related to politics** | When the mistreatment is related to the riot at the U.S. National Capitol, Former President Trump, the Trump campaign, or Trump supporters | “Last president calling it the China virus” “Riot on Capitol”  “Due to Trump, I have always been targeted because I am Asian” |  |
| **Insufficient, unclear, or irrelevant information** |  | Responses that do not include sufficient information, are unclear, are not relevant to the open-ended question or do not indicate experiences of mistreatment | “Everyone” “Everyday” “Yes” “It’s worse” |  |

*All responses could be double coded with themes within the two domains (“type of mistreatment” and “settings where racism occurred”).
